# Diverse Structural Features of Potassium Channels Characterized by Scorpion Toxins as Molecular Probes

**DOI:** 10.3390/molecules24112045

**Published:** 2019-05-29

**Authors:** Yonghui Zhao, Zongyun Chen, Zhijian Cao, Wenxin Li, Yingliang Wu

**Affiliations:** 1 State Key Laboratory of Virology, College of Life Sciences, Wuhan University, Wuhan 430072, China; jimmyzhao@whu.edu.cn (Y.Z.); chenzy2005@126.com (Z.C.); zjcao@whu.edu.cn (Z.C.); wxli@whu.edu.cn (W.L.); 2 Department of Biochemistry and Molecular Biology, Institute of Basic Medical Sciences, College of Basic Medicine, Hubei University of Medicine, Shiyan 442000, China; 3 Biodrug Research Center, Wuhan University, Wuhan 430072, China

**Keywords:** scorpion toxin, potassium channel, channel turret, open channel conformation, half-open or half-closed channel conformation, closed channel conformation

## Abstract

Scorpion toxins are well-known as the largest potassium channel peptide blocker family. They have been successfully proven to be valuable molecular probes for structural research on diverse potassium channels. The potassium channel pore region, including the turret and filter regions, is the binding interface for scorpion toxins, and structural features from different potassium channels have been identified using different scorpion toxins. According to the spatial orientation of channel turrets with differential sequence lengths and identities, conformational changes and molecular surface properties, the potassium channel turrets can be divided into the following three states: open state with less hindering effects on toxin binding, half-open state or half-closed state with certain effects on toxin binding, and closed state with remarkable effects on toxin binding. In this review, we summarized the diverse structural features of potassium channels explored using scorpion toxin tools and discuss future work in the field of scorpion toxin-potassium channel interactions.

## 1. Introduction

**Figure 1:**
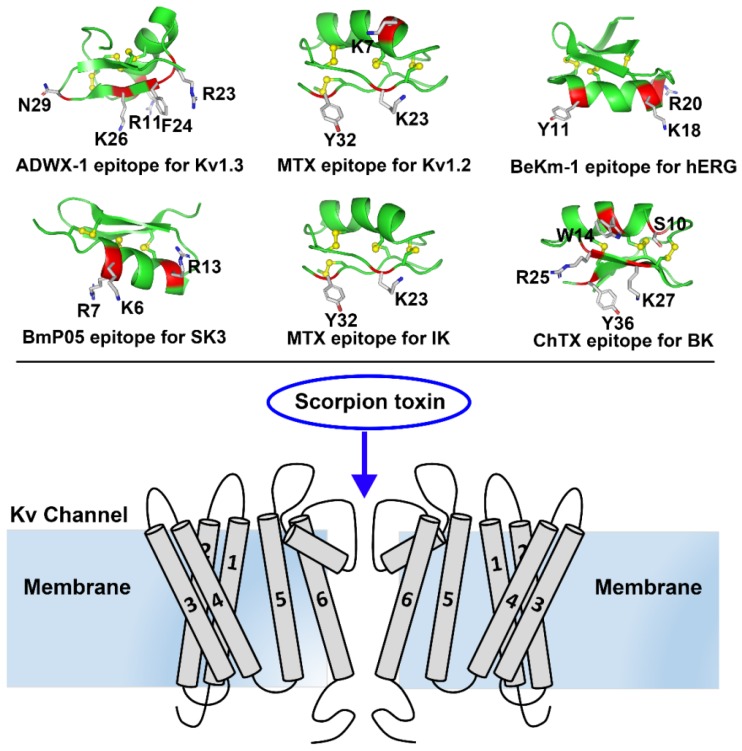
Representative scorpion toxins and toxin-Kv channel interaction diagram. In the upper panel, the representative scorpion toxins with their epitopes: ADWX-1 (PDB ID: 2K4U) epitope for Kv1.3 channel, MTX (PDB ID: 1TXM) epitope for Kv1.2 channel, BeKm-1 (PDB ID: 1LGL) epitope for hERG channel, BmP05 epitope for SK3 channel, MTX (PDB ID: 1TXM) epitope for IK channel, and ChTX (PDB ID: 2CRD) epitope for BK channel. Their roles as the molecular probes of potassium channels will be described in the following sections. In the bottom panel, scorpion toxin works as the molecular probe to block the outer vestibule of channel pore in Kv channels and other type of potassium channels, which can be used to explore the diverse structural features among the different potassium channels.

Potassium channels are a diverse and ubiquitous family of membrane proteins present in both excitable and non-excitable cells. They have been known to be targets of scorpion toxins for approximately 40 years, and investigations focused on the interactions between potassium channels and scorpion toxins have provided a remarkable understanding of the structure and function of diverse potassium channels [1,2,3,4]. Later, the high resolution potassium channel structures obtained by the X-ray crystallography or cryo-electron microscopy have verified the basic features of potassium channels, such as the selectivity filter and turret topology [5,6,7]. However, these limited structures cannot be used to elucidate the sensitivity of potassium channels to numerous scorpion toxins due to channel structural differences, especially in the turret region. A chimaeric KcsA-Kv1.3 potassium channel was found to undergo toxin-induced conformational changes [8]. Thus, scorpion toxins have been used as molecular probes to characterize diverse potassium channels and have played a critical role in understanding the structural and functional diversity of potassium channels. In this review, we review the structural and functional features of representative potassium channels dissected by the representative scorpion toxins (Figure 1).

## 2. Characterization of Potassium Channels

### 2.1. Characterization of Kv1.x Channels

**Figure 2:**
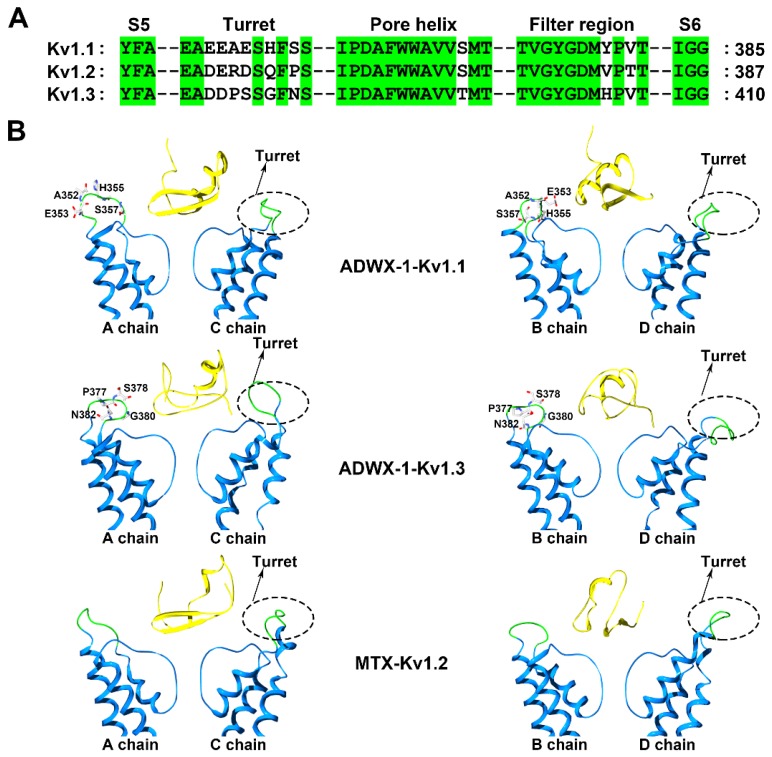
Kv1.1, Kv1.3 and Kv1.2 channel structural features probed by scorpion toxins. (**A**) Amino acid sequence alignment of the Kv1.1, Kv1.2 and Kv1.3 channels. Green shaded letters show identical residues. (**B**) Conformational changes in the Kv1.1, Kv1.3 and Kv1.2 channels induced by the scorpion toxins ADWX-1 (PDB ID: 2K4U) and MTX (PDB ID: 1TXM) [30,37]. The A and C subunits from the Kv1 channels are shown on the left and the B and D subunits from the Kv1 channels are shown on the right. The potassium channel turret region is shown in green and the scorpion toxins are shown in yellow.

Voltage-gated K^+^ (Kv) channels are tetramers, and each subunit comprises six membrane-spanning helical segments, named S1 through S6, as well as three extracellular parts, including the S1-S2 linker, the S3-S4 linker and the pore loop (Figure 1). Scorpion toxins can specifically bind to the pore loop in potassium channels to block channel currents (Figure 1) [9]. The members of the Kv1.x subfamily, especially the Kv1.1, Kv1.2 and Kv1.3 channels, are targeted by many scorpion toxins with differential potencies ranging from the pM to µM [10,11,12,13]. Due to the structural complexity of potassium channels, the exploration of the differential channel conformational responsible for toxin binding has been a challenging task. Potassium channels were found to be blocked by scorpion toxins in the 1980s [14,15]. Using these scorpion toxins as molecular probes, the overall topology of potassium channels was illustrated using site-directed mutagenesis strategies [16,17,18]. This topology was further confirmed by crystal structures of prokaryotic KcsA and mammalian Kv1.2 channels [5,6]. In 2015, a determined complex structure of the scorpion toxin charybdotoxin, from the scorpion *Leiurus quinquestriatus* Var. *hebraeus*, in complex with a Kv1.2-Kv2.1 chimera also indicated that the channel extracellular pore entryway was the toxin binding interface [7]. Superposition of the potassium channel from the charybdotoxin complex onto the channel in the toxin-free structure indicated that the bound channel did not undergo discernible conformational changes at the toxin-binding interface. However, there were obvious kaliotoxin-induced conformational changes in the chimaeric KcsA-Kv1.3 channel revealed by solid-state NMR [8]. These differential observations might suggest different interactions between scorpion toxins and potassium channels. From 2002 to 2015, computational simulations have been widely used to investigate the interactions between scorpion toxins and potassium channels [19,20,21,22,23,24,25,26,27,28], which have provided more structural information to understand scorpion toxin binding towards potassium channels. Most of the computational data were used to explain published work and predict additional interaction details between scorpion toxins and potassium channels, which were not further verified by experimental data. Simultaneously, the diverse structures for the channel extracellular pore vestibule responsible for the selective recognition of scorpion toxins were almost neglected by most researchers during the computational simulations [29]. Using scorpion toxins as molecular probes, the characterization of the different potassium channels was achieved using both experimental and computational data. For example, the scorpion toxin analog ADWX-1 (BmKTX-G11R/I28T/D33H, PDB code: 2K4U) was designed from toxin template BmKTX from the scorpion *Mesobuthus martensii* for treating Kv1.3 channel-related autoimmune diseases (Figure 1). The ADWX-1 has 37 amino acid residues, and adopts a compact fold consisting of an α-helix and antiparallel β-sheet. Such peptide showed a high potency toward the Kv1.3 channel with an IC_50_ value of 1.89 pM [13]. Meanwhile, ADWX-1 also blocked the Kv1.1 channel with an IC_50_ value of 0.65 nM, though it had less of an effect on the Kv1.2 channel. With the help of ADWX-1 as a molecular tool, the structural differences between the Kv1.1 and Kv1.3 channels were characterized. The structural analysis indicated that there is significant similarity in the channel extracellular vestibule, including the turret, pore helix and filter region, between the Kv1.1 and Kv1.3 channels (Figure 2). Using the ADWX-1 toxin as a probe, the residues in the potassium channels that are responsible for the selectivity of ADWX-1 towards Kv1.3 over Kv1.1 were investigated in detail [30]. Through mutagenesis and computational experiments, the Kv1 channel turret, but not the filter region, was found to be responsible for the high selectivity of the ADWX-1 peptide for Kv1.3 over Kv1.1 channels. This observation was different from the selective binding of kaliotoxin and charybdotoxin towards Kv1.x channels, in which a variable residue in the Kv1.x channel pore region (Tyr379 in the Kv1.1 and His404 in the Kv1.3 channels) (Figure 2A) was critical for the high affinity of scorpion toxins [31]. These findings were also in line with the indiscernible conformational changes between the toxin-binding and toxin-free potassium channels [7]. Certainly, these differential roles of the channel pore regions in scorpion toxin binding were likely caused by the scorpion toxins using different functional residues to bind potassium channels. When ADWX-1 associated with the potassium channel, a mutant of the Kv1.1 channel (Kv1.1-AEHS/PSGN), in which four key residues in the Kv1.1 turret were simultaneously replaced with the corresponding residues in Kv1.3 turret (Figure 2B), had a similar sensitivity to ADWX-1 as the Kv1.3 channel. ADWX-1 blocked the Kv1.1-AEHS/PSGN channel with an IC_50_ of 3.94 pM, which was comparable to ADWX-1′s potency for the Kv1.3 channel [30]. Interestingly, the single residue substitution mutant channels, including the Kv1.1-A352P, Kv1.1-H355A and Kv1.1-S357N channels, showed much less sensitivity to ADWX-1 than the Kv1.3 channel. The IC_50_ values for ADWX-1 for the Kv1.1-A352P, Kv1.1-H355A, and Kv1.1-S357N mutants were 1.38, 0.15, and 0.55 nM, respectively [30]. The remarkable sensitivity differences between the mutant Kv1.1 channels from the single and combined residue replacement demonstrated that the different residues in the channel turrets play essential roles in binding scorpion toxins through synergetic interactions with scorpion toxins. The subsequent channel-toxin complex structures from the computational simulations indicated that the channel turrets from one diagonal chain make close and differential contacts with ADWX-1 toxin, while the channel turrets from the other diagonal chains bent outward and were far away from the ADWX-1 toxin (Figure 2B) [30]. Overall, the different conformational arrangements of channel turrets and the overall weaker interactions between the Kv1.1 channel and the ADWX-1 toxin contributed to the lower binding affinity for the ADWX-1 peptide than by the Kv1.3 channel.

Interestingly, the channel turret does not always determine the sensitivity of Kv1.x channels towards scorpion toxins. This sensitivity predominantly depends on the filter region conformation. The *Scorpio maurus palmatus* scorpion toxin maurotoxin (MTX, PDB code: 1TXM) has 34 amino acid residues, and adopts a compact fold consisting of an α-helix and antiparallel β-sheet (Figure 1). During the binding of the Kv1.2 channel by the toxin MTX, which has an IC_50_ value of 0.7 nM [32], a three residue substitution of Arg354, Glu355 and Asp363 in the Kv1.2 channel turret hardly altered MTX affinity. Using computational simulations, the four turrets in the Kv1.2 channel were found to bend outwards and stay far away from the scorpion toxin MTX, which could explain the lesser effects of residue replacement in the Kv1.2 channel turret (Figure 2B) [33]. The residues in the Kv1.2 channel pore region were responsible for MTX binding. The MTX-induced conformational changes in the Kv1.2 channel also explained the interaction between the Kv1.3 channel and MTX (Figure 2B). The Kv1.3 channel is not sensitive to MTX binding, displaying an IC_50_ value of 3.3 μM. In the “GYGDMH” motif of the channel filter region, a histidine substitution by threonine led to an increased potency of MTX towards Kv1.3 mutant (IC_50_ value of 0.6 nM) [32]. In contrast, the turret is important for Kv1.3 channel binding to the scorpion toxin ADWX-1 [13,30], as the Kv1.3 channel can change the roles of the turret and filter region upon different scorpion toxin binding.

Besides the Kv1.2 channel filter region responsible for the binding of scorpion toxin MTX, it was also found that such domain could determine the sensitivity of Kv1.2 channel towards other scorpion toxins. The mesomartoxin showed differential selectivity on Kv1.x channels with nanomolar affinity (IC_50_ = 15.6 nM) for rat Kv1.2 channel, micromolar affinity (IC_50_ = 12.5 μM) for rat Kv1.3 channel and no activity on rat Kv1.1 channel at > 50 μM [34]. Based on sequence diversity, chimeras were built to identify which residues were responsible for toxin binding at the pore filter/turret region. The authors respectively constructed five Kv1.1 channel mutants Kv1.1-A352R, Kv1.1-H355Q, Kv1.1-A352R/H355Q, Kv1.1-S357P, Kv1.1-A352R/H355Q/S357P according to the differential residues between Kv1.1 and Kv1.2 turrets, and 2 mutants Kv1.1-Y379V and Kv1.1-V381T based on the differential residues between Kv1.1 and Kv1.2 filter regions. Among these Kv1.1 channel mutants, the electrophysiological experiments indicated that only Kv1.1-Y379V mutant channel was sensitive towards scorpion toxin mesomartoxin with IC_50_ value of 16.6 nM, which was comparable to that of the wild-type Kv1.2 channel [34]. These investigations demonstrated that the filter region of Kv1.2 channel was critical for mesomartoxin binding.

Recently, the importance of Kv1.2 channel filter region for scorpion toxin binding was also verified by the *Mesobuthus eupeus* scorpion toxins MeKTx11-1 and its analogs [35]. Toxins MeKTx11-1, MeKTx11-3, MeKTx11-1(G9V) and MeKTx11-1 (P37S) could selectively block Kv1.2 channel with IC_50_ values of 0.19 nM, 3.1 nM, 0.07 nM and 0.09 nM, respectively, while their activities against the second sensitive Kv1.3 channels were 67 nM, 78 nM, 1.4 nM and 13.6 nM, respectively. In order to understand which differential channel residues were responsible for scorpion toxin selectivity, two Kv1.3 channel mutants Kv1.3-D376E/P377R/T378D/G380Q/S382P and Kv1.3-T378D/G380Q were constructed according to the differential residues between Kv1.2 and Kv1.3 channel turrets, and the third mutant Kv1.3-H404V/V406T was designed based on the differential residues between Kv1.2 and Kv1.3 channel filter regions. Overall, the pharmacological profiles of Toxins MeKTx11-1, MeKTx11-3, MeKTx11-1(G9V) and MeKTx11-1 (P37S) indicated that the Kv1.3-D376E/P377R/T378D/ G380Q/S382P and Kv1.3-T378D/G380Q mutant channels did not become more remarkably sensitive towards scorpion toxins when these residues in the Kv1.3 channel turret were substituted by the corresponding residues in the Kv1.2 turret [35]. However, the sensitivities of Kv1.3-H404V/V406T mutant channel towards MeKTx11-1, MeKTx11-3, MeKTx11-1(G9V) and MeKTx11-1 (P37S) were significantly improved when His404 and Val406 in the Kv1.3 channel filter region were replaced by the corresponding Val377 and Thr379 in the Kv1.2 channel filter region. These works also revealed that the filter region of Kv1.2 channel instead of channel turret was essential for the binding of scorpion toxins MeKTx11-1 and its analogs.

Clearly, toxin selectivity among Kv1.x channels remains a challenge currently and for the foreseeable future. More work, especially on the experimental determination of complex structures, should be completed to investigate the differential roles of the turret and filter regions from Kv1.x channels for an in-depth understanding of the channel conformational change features. This structural information would be helpful to discover and/or design scorpion toxin-derived peptide drugs for Kv1.3 channel-associated autoimmune diseases [36].

### 2.2. Characterization of the hERG Channel

Among the superfamily members of potassium channels, the human ether-a-go-go-related gene (hERG) potassium channel plays an essential role in mediating the process of returning the membrane potential to its resting status. An inherited mutation in the normal human hERG gene can cause long-QT syndrome-related proarrhythmia and sudden death. Moreover, a similar disorder called acquired-long QT syndrome can be triggered by drugs via blockage of the hERG channel. Thus, hERG has now become a focus target in the pharmaceutical industry for detecting this undesirable side effect.

**Figure 3:**
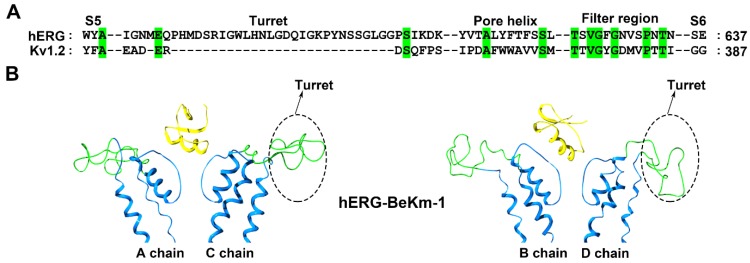
hERG channel structural features. (**A**) Amino acid sequence alignment of the hERG and Kv1.2 channels. Green shaded letters show identical residues. (**B**) Conformational changes in the hERG channel induced by the scorpion toxin BeKm-1 (PDB ID: 1LGL) [39]. The A and C subunits in the hERG channel are shown on the left and the B and D subunits in the hERG channel are shown on the right. The potassium channels turret region is shown in green and the scorpion toxins are shown in yellow.

Different from the classical Kv1.x potassium channels, the hERG channel has an unusual longer turret containing 40 amino acid residues (Figure 3A), and such turret structure was shortage in a recent cryo-EM structure of the hERG channel [38]. The *Buthus eupeus* scorpion toxin BeKm-1 has 36 amino acid residues, and adopts a compact fold consisting of an α-helix and antiparallel β-sheet (Figure 1). Using the toxin BeKm-1 as a molecular probe, different conformational changes in the four turrets of the hERG channel were reported [39,40]. Using computational simulation techniques, an open conformation was proposed where the four decentralized turrets formed a “petunia”-like shape far from where the toxin BeKm-1 bound (Figure 3B) [39]. Meanwhile, another model without a “petunia” shape was also predicted, in which the four channel turrets kept erect and directly interacted with the bound BeKm-1 based on cysteine-scanning mutagenesis and computational modeling [40]. Actually, cysteine substitutions in the hERG channel turrets at different positions were found to have high-, intermediate- or low-impact on channel functions and affect scorpion toxin binding due to the potential formation of disulfide bonds between the channel cysteine and introduced cysteine amino acid residues [40,41].

To clarify the structural features of the hERG channel turrets in scorpion toxin binding, the scorpion toxin BmKKx2, which shares nearly identical functional residues to those in the homologous BeKm-1 toxin, was used as a molecular probe [42]. An alanine-scanning mutagenesis strategy was adopted to avoid the influence of the disulfide bonds between the channel cysteines and introduced cysteine amino acid residues. Using 40 residues in the hERG channel turret, 20 mutant channels were constructed for the in-depth investigation of the structural channel turret features in scorpion toxin binding [42]. In line with the computational simulation between the hERG channel and the scorpion toxin BeKm-1 (Figure 3B) [39], alanine-scanning substitution in the hERG channel turret indicated that the turrets played fewer effects on scorpion toxin binding due to their forming an open conformation [42]. Thus, it was understandable that the conformation of the hERG channel filter region significantly affected scorpion toxin binding. The channel Ser631 residue near the hERG channel selectivity filter was found to be essential for BmKKx2 binding, as shown by the approximately 104-fold drop in affinity in the hERG-S631A channel compared with the wild-type hERG channel [42]. In the cysteine-scanning experiments [40], the importance of the Ser631 residue in the hERG channel was less significant; its substitution by cysteine was found to have an intermediate-impact on hERG channel function as shown by the differential effects in the mutant channel function in the presence or absence of the reducing agent DTT [41]. However, why the hERG channel turrets formed an open conformation remains to be answered.

These studies demonstrated that experiments involving cys mutagenesis should be considered with precaution, especially when there are channel cysteine residues spatially adjacent to the target residues in the channel turret and filter region. Otherwise, there might be risk when rationally evaluating potassium channels.

From the recent cryo-EM structure of the hERG channel, it could be seen that four short helices in the channel turrets formed a much narrow entrance for the scorpion toxins [38], which suggested the channel turret would likely play an important role in the binding of scorpion toxins. However, such importance was not found by the extensive alanine-scanning mutagenesis in the hERG channel turret, which indicated that the turret structures played fewer effects on scorpion toxin [42]. The differential comformations of hERG channel turret between cryo-EM and modeled structures of the hERG channel implied that the flexible hERG channel turret dynamically changed with or without the scorpion toxin binding [38,39], which would be clarified by determining the scorpion toxin-hERG channel complex structure in the future.

### 2.3. Characterization of SK Channels

Small conductance calcium-activated (SK) channels are predominantly present in the mammalian nervous system. They are involved in synaptic plasticity, fast glutamatergic synaptic potentials, and hippocampal learning [43,44,45]. SK channels are efficiently blocked by several scorpion toxins, such as BmP05, scyllatoxin and P05, but are insensitive towards many other scorpion toxins [46,47]. Undoubtedly, this specific recognition depends on the unusual structural feature of the pore region in SK channels.

**Figure 4:**
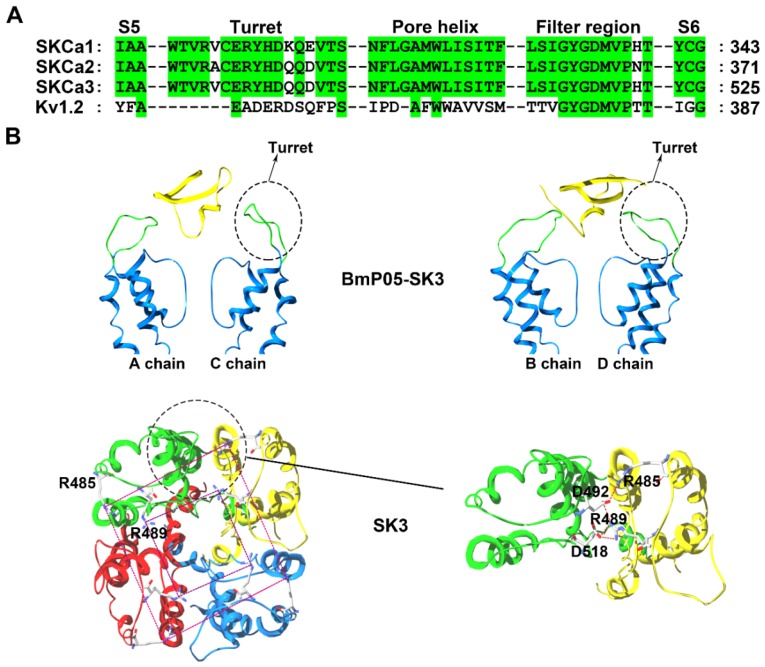
SK channel structural features. (**A**) Amino acid sequence alignment of the SK and Kv1.2 channels. Green shaded letters show identical residues. (**B**) The peptide screener conformation of the SK3 channel [50]. In the upper panel, the A and C subunits in the SK3 channel are shown on the left and the B and D subunits in the SK3 channel are shown on the right. The potassium channel turret region is shown in green and the scorpion toxin BmP05 are shown in yellow. In the bottom panel, top view of the narrow gateway in the SK3 channel turrets. The large and small rings of basic residues are designated by different color dashed lines, respectively.

Although nonclassical turrets from SK channels with longer sequence lengths than the Kv1.2 channel were proposed earlier (Figure 4A) [29], this feature was neglected when modeling SK channels with the same turret sequence length as the Kv1.2 channel in previous works [19,48]. It is actually difficult to model a target protein whose sequence is longer than that of the template protein. Therefore, the subsequent modeling of IK and BK channels also encountered the same problem during the computational simulations of scorpion toxin-potassium channel interactions [22,49]. In 2007, a segment-assembly homology modeling method was developed [39], and more reasonable modeling of SK, IK and BK channels was achieved for understanding their structural features.

The structural analysis indicated that SK channels are selectively inhibited by specific scorpion toxins containing no more than three basic amino acid residues in their channel binding interfaces, which was experimentally confirmed by scorpion toxins with more or less than three basic residues in their channel binding interfaces [50]. For example, the *Mesobuthus martensii* scorpion toxin BmP05, a 31-residue peptide, can efficiently block SK3 channel currents with an IC_50_ value of 3.8 nM (Figure 1). However, increasing in the number of basic residues by one (at SK3 channel-sensitive toxin BmP05 position 14 or 15) or two arginine residues (at BmP05 positions 4 and 14 or 4 and 15), significantly reduced the BmP05 blockage potency. This selective SK channel recognition was determined by a possible compact turret conformation, which was named by the peptide screener (Figure 4B) [50,51]. In the SK3 channel, the modeled peptide screener was formed by two basic rings created by conserved Arg485 and Arg489 residues (Figure 4B). The differential electrostatic repulsion forces between the basic rings in the SK3 channel and the basic residues at the channel-binding interfaces for scorpion toxins could govern the selective binding of the positively charged scorpion toxin peptides. To maintain the stability of peptide screener in the SK3 channel, both Arg485 and Arg489 formed salt bridges with aspartic acid residues from adjacent SK3 subunits (Arg485 with Asp492 and Arg489 with both Asp492 and Asp518) (Figure 4B) [50]. When the scorpion toxin BmP05 approached the SK3 channel, the Lys6 from BmP05 blocked the channel pore and Arg7 from BmP05 was located between two Arg489 residues from the peptide screeners; in contrast, Arg13 from the toxin hung just above the two basic rings from the peptide screener. This contact mode could likely minimize the electrostatic repulsion forces between BmP05 and the SK3 channel [50]. Scorpion toxins with a greater number of basic amino acid residues at the channel-interacting surface were unable to recognize the SK3 channel owing to stronger electrostatic repulsion forces between the toxins and basic rings from the peptide screener.

The toxin recognition profile of SK3 channels (sensitive to BmP05-like toxins and insensitive to charybdotoxin-like toxins) could be maintained by basic amino acid substitutions in the peptide screener, but inverted by acidic or polar amino acid residue replacements [50]. Basic amino acid substitutions resulted in reduced BmP05 blockage effects. Dose response experiments indicated reduced affinity by the mutant channels for BmP05 by 88-fold (SK3-R485H), 101-fold (SK3-R489H), 38-fold (SK3-R485K), and 51-fold (SK3-R489K), which were based on the basic nature of the basic residues in the peptide screener [50]. Clearly, this degree of reduction in BmP05′s effect was related to the stability of the peptide screener conformation. When both the Arg485 and Arg489 residues were replaced by the negative residues, the peptide screener displayed a looser conformation as a consequence of the weaker electrostatic interactions between the four SK3 channel subunits. The looser turret structure further impaired the interactions between scorpion BmP05 and the SK3 channel. Once the compact conformation of the peptide screener was lost by replacing both Arg485 and Arg489 with acidic or polar residues, the mutant SK3 channels became insensitive to the scorpion toxin BmP05 and were significantly blocked by charybdotoxin. The dose response experiments showed IC_50_ values for the charybdotoxin-induced current blockage of 381 nM (SK3-R485E), 110 nM (SK3-R489E), 84 nM (SK3-R485S), and 30 nM (SK3-R489S) [50]. In line with the structural stability of the peptide screener for the SK channel-sensitive BmP05-like toxins and the substitutions of both Arg485 and Arg489 by acidic or polar residues, the critical salt bridge interactions between Arg485 and Arg489 in the peptide screener and the aspartic acid residues from adjacent SK3 subunits would be disrupted, which made the mutant SK3 channel insensitive to BmP05 toxin but sensitive to the wild-type SK channel-insensitive charybdotoxin [50].

In the SK channel subfamily, structural analysis indicated that both the Arg485 and Arg489 residues are conserved among members. In view of their similar pharmacological features, the peptide screener becomes a common structural determinant with regard to its selective recognition profile for scorpion toxins [51].

Apart from the SK channel turret, the conformation of channel filter region also seriously affected the toxin binding. For example, the SK3-D518N was found much less sensitive to scorpion toxin BmP05 compared with the wild-type SK3 channel [50]. Such observations demonstrated that both the turret and filter region structures of SK3 channel were responsible for scorpion toxin binding.

### 2.4. Characterization of the IK Channel

The intermediate-conductance calcium-activated potassium channel (IK) acts as a modulator of cell proliferation by hyperpolarizing the cell membrane in T and B cells, fibroblasts and vascular smooth muscle cells [52,53,54]. The scorpion toxin MTX is a potent blocker of the IKCa channel (Figure 1) [32,55]. Using MTX as a molecular tool, structural characterization was performed using experimental and computational strategies [32,37,55].

**Figure 5:**
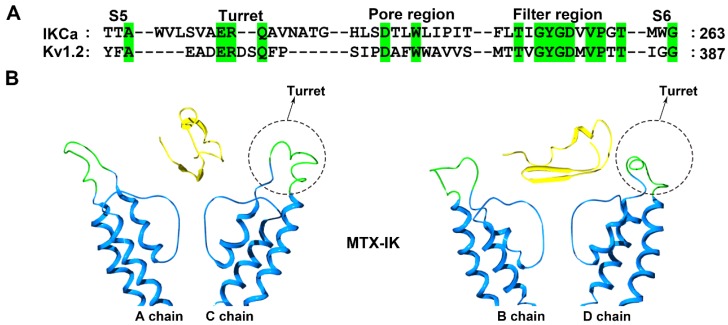
IK channel structural features. (**A**) Amino acid sequence alignment of the IK and Kv1.2 channels. Green shaded letters show identical residues. (**B**) Conformational changes in the IK channels induced by the scorpion toxin MTX (PDB ID: 1TXM) [37]. The A and C subunits in the IK channel are shown on the left and the B and D subunits in the IK channel are shown on the right. The potassium channel turret region is shown in green and the scorpion toxins are shown in yellow.

Structurally, the IK channel has a longer turret region than the Kv1.2 channel, which is also efficiently blocked by MTX (Figure 5A). Due to the lower sequence identity between the channel turrets, there are some differences in the MTX channel-interacting interface [32,55]. For example, the replacement of toxin Lys7 by an alanine residue could decrease the toxin affinity by approximately 100-fold in the case of the Kv1.2 channel, but the decrease was less than 10-fold in the case of the IKCa channel [32,55]. The computational simulation analysis indicated that the four IK channel turrets played similar roles in recognizing MTX to those in the Kv1.1 and Kv1.3 channel turrets for binding the toxin ADWX-1 (Figure 2 and Figure 5). The turrets from one diagonal channel chain interacted well with MTX, and several residues, including Gln229, Ala230, Ala233 and Thr234, in the IKCa turret formed polar and nonpolar interactions with the spatially adjacent residues in MTX [37]. However, the turrets from the other diagonal channel chains remained far away from MTX (Figure 5B). These toxin-induced conformational features were different from the Kv1.2 channel, whose four turrets were almost not involved in recognizing MTX [25]. These observations showed that potassium channels could adjust their own preferred conformations for specifically binding the same scorpion toxin.

### 2.5. Characterization of the BK Channel

The large conductance Ca^2+^- and voltage-dependent potassium (BK) channel presents its functional diversity mainly caused by the co-expression of pore-forming α subunits with tissue-enriched auxiliary β subunits [56,57,58,59]. This functional diversity is also verified by the differential blockage effects of scorpion toxins. For example, the α + β1 channel shows charybdotoxin (ChTX) sensitivity similar to that of an α channel (Figure 1) [57]. However, BK channels associated with the β2 or β3 subunits usually have approximately 30-fold lower sensitivity to ChTX [57,58], whereas the α + β4 channel has approximately 1000-fold lower sensitivity to the same toxin [59]. Clearly, there are various structural features of scorpion toxin binding sites in BK channels from the different complexes formed by one α and four different β subunits.

**Figure 6:**
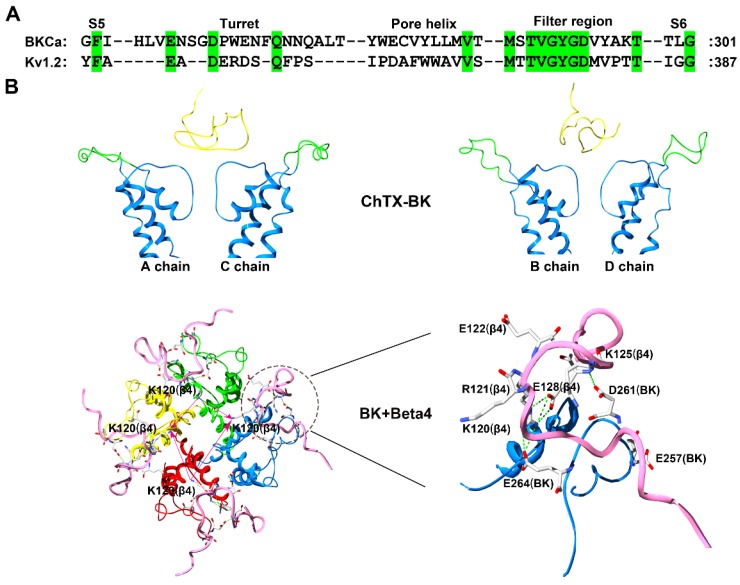
BK channel structural features. (**A**) Amino acid sequence alignment of the BK (α subunit) and Kv1.2 channels. Green shaded letters show identical residues. (**B**) Conformational characterization of the BK channel probed by the scorpion toxin ChTX (PDB ID: 2CRD) [63]. The A and C subunits in the BK channel are shown on the left and the B and D subunits in the BK channel are shown on the right. The potassium channel turret region is shown in green, the scorpion toxins are shown in yellow, and the β4 subunit is shown in pink.

The ChTX was used to characterize BK channels only containing α subunits based on experimental and computational data [60,61,62,63]. The ChTX has 37 amino acid residues, and adopts a compact fold consisting of an α-helix and antiparallel β-sheet (Figure 1) (PDB code: 2CRD). Similar to the conformational features of hERG channels, the four turrets from unbound BK channels likely disperse one another from the channel pore region symmetric axis (Figure 6A) [62,63]. In the bound BK channel with ChTX, the four turrets had fewer effects on the toxin binding affinity, as they were far away from ChTX (Figure 6B).

Next, conformational changes in the four channel turrets were further investigated in the α + β4 channel, which presents the unusual toxin resistance [59]. According to the special peptide screener from SK channels responsible for the selective recognition of scorpion toxins [50,51], the positively charged residues in the extracellular loop of the β4 subunit were also found to be essential for toxin resistance [63]. In comparison with the insensitivity of the α + β4 channel towards ChTX, the simultaneous replacement of Lys120 and Arg121 with alanine and asparagine acid residues, respectively, in the middle of the β4 subunit extracellular loop made the mutant channel sensitive to ChTX with a K_d_ value of 27.1 nM, which was 8.2-fold lower than that of the toxin when blocking BK channels without β subunits [63]. Additionally, the substitution of Lys125 with a threonine residue in the β4 subunit could make the mutant channel sensitive to ChTX with a K_d_ value of 15.1 nM. This loss and restoration of channel sensitivity to ChTX between the wild-type and mutant BK channels depended on the conformational change in the special “helmet” structure in the ChTX-binding region in the α + β4 channel (Figure 6B). In the wild-type α + β4 channel, basic residues Lys120, Arg121 and Lys125 in the β4 subunit like to form salt-bridges with acidic residues Glu257, Asp261 and Glu264 in the α subunit turret (Figure 6B). These strong electrostatic interactions prevent the four turrets from being far from the channel pore region symmetric axis. The three the basic residues have double functional roles in the helmet structure, as one is important for the structural stability of the helmet through electrostatic attraction with acidic residues in the α subunit turret and the other is for scorpion toxin resistance through the electrostatic repulsion with the toxin basic residues (Figure 6B).

The effect of the helmet-like structure in the α + β4 channel appears to be a common structural feature of other BK channel subtypes. The substitution of basic residues in the β2 subunit extracellular loop similarly enhanced the sensitivity of the α + β2 channel to ChTX [63]. These observations could also explain why the α + β1 channel shows ChTX sensitivity similar to the α channel, as there are fewer basic residues in the β1 subunit extracellular loop [57,63]. Altogether, the scorpion toxin can be used to probe more complex structures in potassium channels.

## 3. Concluding Remarks and Future Directions

**Figure 7:**
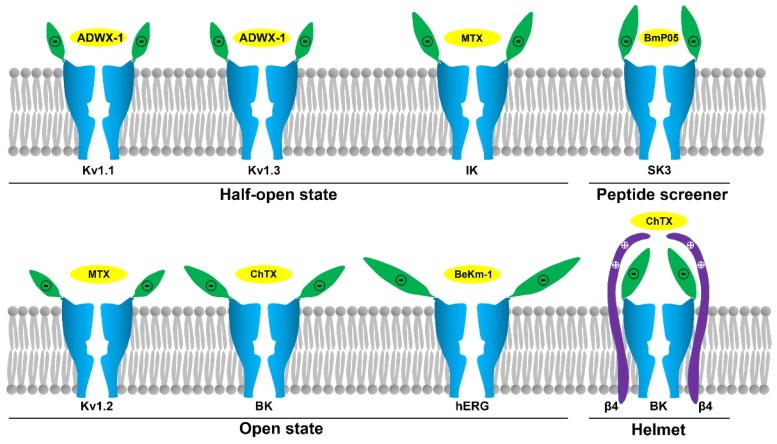
Structural diversity of potassium channels explored by scorpion toxins. The half-open state in the Kv1.1, Kv1.3 and IK channels; the open state in the Kv1.2, hERG and BK (only α subunit) channels; the half open-like state (peptide screener) in the SK channels; and the closed state (helmet) in the BK (α + β4) channel [30,33,37,39,42,50,51,63]. The potassium channel turret region is shown in green and the scorpion toxins are shown in yellow.

It is well-known that scorpion toxins constitute the largest potassium channel peptide blocker family. In the past decades, these toxins have been successfully proven to be valuable molecular probes for channel structural and functional research, especially for Kv1.x, hERG, SK, IK and BK channels (Figure 1). These studies highlight the structural and functional diversity of potassium channel pore regions, which includes their sequence length, sequence identity, turret conformation, and molecular surface properties. These factors jointly determine their sensitivity towards different scorpion toxins. Current investigations have shown the various conformational states in the pore region of bound potassium channels explored by scorpion toxins, including the half-open state in Kv1.1, Kv1.3 and IK channels; the open state in Kv1.2, hERG and BK (only α subunit) channels; the peptide screener in SK channels; and the helmet in BK (α + β4) channels (Figure 7). These various channel turret conformations induced by scorpion toxin binding could be regarded as mysterious and beautiful flowers with diverse shapes, including a bud resistant to working bees for the scorpion toxin, alabastrum-specific for the small bee, and half-open and open flower for an ordinary bee. Certainly, the different channel turrets work as different petals, and different bees represent different scorpion toxins with different sequence lengths and identities. It should be noted that some of these channel conformational states are likely flexible, especially for Kv1.x, IK and BK (only α subunit) channels, whose conformations are likely to change when binding different scorpion toxins. However, some of above channel conformational states are stable, especially the peptide screener in SK channels and the helmet in BK (α + β4) channels, because the salt bridge interactions are sufficient to maintain these special structures. It is noted that these various channel turret conformations from the computational simulations still represent possible trends of channel conformational changes (Figure 7), although they could explain the selectivity of scorpion toxins at various degrees. It is known that there are certain differences between the computational simulations and biological experiments on the protein-protein interactions since the optimization of protein constraints in chemical and solvent properties is still advancing during the computational simulations. Due to the conformational flexibility of potassium channel turrets, it is still expected that more high resolution structures will be determined in the future.

With the wide application of genomic, transcriptomic, and proteomic approaches, more and more scorpion toxins have been found [64,65,66,67,68,69,70]. In 2004, there were approximately 120 known scorpion toxins [46]; currently, the number has risen to approximately 550 scorpion toxins (data from the UniProt database, http://www.uniprot.org/). Unfortunately, the pharmacological profiles of most of these scorpion toxins remain unknown. Similarly, sensitive spectra for most potassium channel subtypes towards scorpion toxins also remain unknown. Therefore, future work should be focused on the following areas: (1) More investigations on the interactions between scorpion toxins and potassium channels, especially for potassium channels without toxin probes; (2) The conformational features of a specific potassium channel bound by different scorpion toxin probes; and (3) Structural determination of representative scorpion toxin-potassium channel complexes explored by X-ray crystallography or cryo-electron microscopy. These studies would be helpful for finding novel interactions between scorpion toxins and potassium channels, as well as for characterizing novel features of channel conformational changes, which will promote a better understanding of the structure-function relationships of diverse potassium channels and the discovery of potassium channel-specific blockers as more efficient molecular probes or drug leads.
